# Gastric and rectal administration of encorafenib with targeted chemotherapy against BRAF V600E–mutant rectal cancer with bowel obstruction

**DOI:** 10.1093/oncolo/oyag103

**Published:** 2026-03-31

**Authors:** Maximilian Alexander Funk, Volker Heinemann, Veit Bücklein, Stefan Karl Alig, Kathrin Heinrich, Lena Weiss, Victoria Krenmayr, Benoit Blanchet, Wolfgang Gerhard Kunz, Sebastian Theurich, Michael von Bergwelt-Baildon, Julian Walter Holch

**Affiliations:** Department of Medicine III, LMU University Hospital Munich, Munich, 81377, Germany; Comprehensive Cancer Center Munich, Munich, 81377, Germany; German Cancer Consortium (DKTK), Partner Site Munich and German Cancer Research Centre (DKFZ), Heidelberg, Munich, 80336, Germany; Bavarian Cancer Research Center (BZKF), Munich, 81377, Germany; Comprehensive Cancer Center Munich, Munich, 81377, Germany; Department of Medicine III, LMU University Hospital Munich, Munich, 81377, Germany; Comprehensive Cancer Center Munich, Munich, 81377, Germany; Bavarian Cancer Research Center (BZKF), Munich, 81377, Germany; Department of Hematology and Stem Cell Transplantation, West German Cancer Center, University Hospital Essen, University of Duisburg-Essen, Essen, 45147, Germany; German Cancer Consortium (DKTK), Partner Site Essen and German Cancer Research Centre (DKFZ), Heidelberg, Essen, 45147, Germany; Department of Medicine III, LMU University Hospital Munich, Munich, 81377, Germany; Comprehensive Cancer Center Munich, Munich, 81377, Germany; German Cancer Consortium (DKTK), Partner Site Munich and German Cancer Research Centre (DKFZ), Heidelberg, Munich, 80336, Germany; Bavarian Cancer Research Center (BZKF), Munich, 81377, Germany; Department of Medicine III, LMU University Hospital Munich, Munich, 81377, Germany; Comprehensive Cancer Center Munich, Munich, 81377, Germany; German Cancer Consortium (DKTK), Partner Site Munich and German Cancer Research Centre (DKFZ), Heidelberg, Munich, 80336, Germany; Bavarian Cancer Research Center (BZKF), Munich, 81377, Germany; Department of Medicine III, LMU University Hospital Munich, Munich, 81377, Germany; Comprehensive Cancer Center Munich, Munich, 81377, Germany; German Cancer Consortium (DKTK), Partner Site Munich and German Cancer Research Centre (DKFZ), Heidelberg, Munich, 80336, Germany; Bavarian Cancer Research Center (BZKF), Munich, 81377, Germany; Service de Biologie du Médicament et Toxicologie, CARPEM, Hôpital Cochin, AP-HP, Paris, Paris, 75014, France; Université Paris Cité, CNRS, INSERM CiTCoM, Paris, 75006, France; Department of Radiology, LMU University Hospital Munich, Munich, 81377, Germany; Department of Medicine III, LMU University Hospital Munich, Munich, 81377, Germany; Comprehensive Cancer Center Munich, Munich, 81377, Germany; German Cancer Consortium (DKTK), Partner Site Munich and German Cancer Research Centre (DKFZ), Heidelberg, Munich, 80336, Germany; Bavarian Cancer Research Center (BZKF), Munich, 81377, Germany; Department of Medicine III, LMU University Hospital Munich, Munich, 81377, Germany; Comprehensive Cancer Center Munich, Munich, 81377, Germany; German Cancer Consortium (DKTK), Partner Site Munich and German Cancer Research Centre (DKFZ), Heidelberg, Munich, 80336, Germany; Bavarian Cancer Research Center (BZKF), Munich, 81377, Germany; Department of Medicine III, LMU University Hospital Munich, Munich, 81377, Germany; Comprehensive Cancer Center Munich, Munich, 81377, Germany; German Cancer Consortium (DKTK), Partner Site Munich and German Cancer Research Centre (DKFZ), Heidelberg, Munich, 80336, Germany; Bavarian Cancer Research Center (BZKF), Munich, 81377, Germany

**Keywords:** metastatic colorectal cancer, BRAF mutation, peritoneal carcinomatosis, encorafenib, bowel obstruction

## Abstract

The activating BRAF mutation V600E occurs in 8%–12% of metastatic colorectal cancers (mCRC) and is associated with peritoneal carcinomatosis (PC) and poor prognosis. Targeted inhibition with the oral BRAF inhibitor encorafenib, combined with intravenous cetuximab targeting epithelial growth factor receptor (EGFR) and standard chemotherapy FOLFOX, has improved outcomes and received FDA approval in 2025 based on results from the phase III BREAKWATER trial. We report a 26-year-old male with rapidly progressive, BRAF V600E-mutant, microsatellite-stable (MSS) rectal adenocarcinoma, liver metastases, and PC presenting with bowel obstruction and inability for oral intake. To enable targeted therapy, encorafenib was administered rectally and via nasogastric tube, together with intravenous cetuximab and FOLFOX (FOLFOX+EC). Most adverse events (AEs) were present before treatment and improved during FOLFOX+EC, indicating tumor association. Severe AEs included emesis, fatigue, pain, and elevated cholestatic liver enzymes. Treatment-related AEs were manageable with anaemia (°3), neutropenia (°4), and thrombocytopenia (°1). No encorafenib-specific toxicities (rash, pyrexia, QTc prolongation, or ocular effects) were observed. Pharmacokinetic analysis showed trough plasma exposure comparable to oral dosing in patients without PC, and CT imaging at weeks 4 and 8 revealed early tumor shrinkage. This case demonstrates that rectal and nasogastric administration of encorafenib is feasible, achieves therapeutic plasma concentrations, and induces objective and clinical remission in the context of FOLFOX+EC. Short-term safety appeared manageable, though increased infection risk cannot be excluded.

Key Points

- BRAF^V600E^ mutant CRC is associated with an increased incidence of peritoneal carcinomatosis and an overall worse prognosis.
- Targeted inhibition of BRAF^V600E^ in combination with inhibition of EGFR in combination with chemotherapy is an effective therapeutic approach but requires oral administration of encorafenib, which can be challenging in the presence of bowel obstruction.
- We present the case of a patient with rapidly progressing metastatic BRAF^V600E^ mutant rectal adenocarcinoma and bowel obstruction. Gastric and rectal administration of encorafenib was safe and resulted in therapeutic serum concentrations and clinical response.

## Introduction

Activating mutation V600E of the oncogene v-Raf murine sarcoma viral oncogene homolog B1 (BRAF) occurs in 8%-12% of newly diagnosed metastatic colorectal carcinomas (mCRC), defining a distinct molecular subtype.^1^^,^^2^ Here, the presence of a BRAF^V600E^ mutation is associated with peritoneal carcinomatosis (PC) and confers a worse prognosis compared to BRAF wild-type tumors.^3–5^ A distinct obstacle in the context of PC is bowel obstruction that impedes oral drug administration. In this study, we report the case of a patient with a BRAF V600E (BRAF^V600E^)-mutant rectal adenocarcinoma and synchronous hepatic and peritoneal metastasis. Due to subtotal bowel obstruction, encorafenib was applied rectally and via nasogastric tube in combination with intravenous cetuximab and chemotherapy.

## Patient story

**Figure 1.**
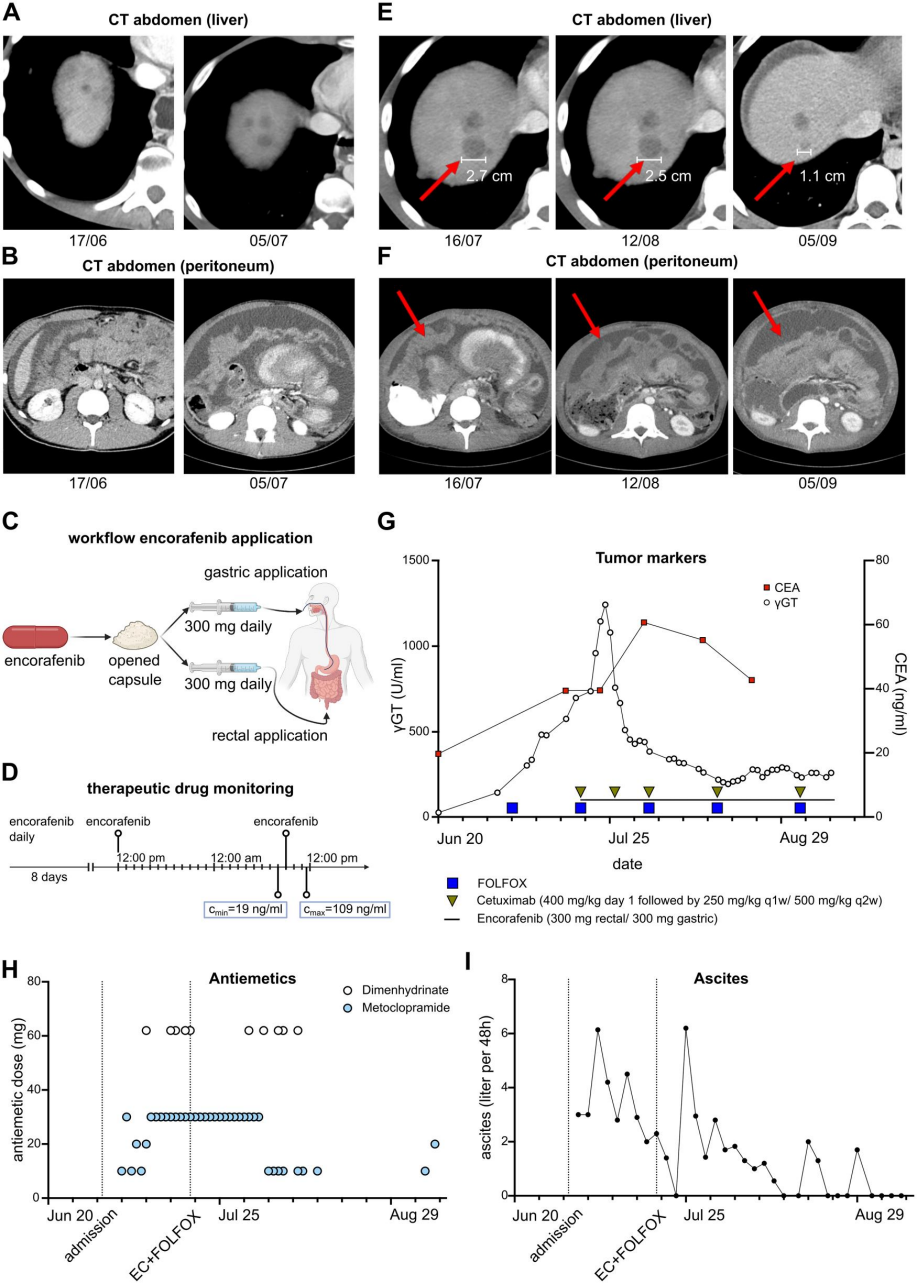
(A, B) CT scans of liver (A) and abdomen (B) show rapidly progressing hepatic and peritoneal lesions prior to therapy. (C) Scheme indicating the workflow of gastric and rectal application of encorafenib. (D) Scheme depicting time course of encorafenib therapeutic drug monitoring. (E, F) CT scans document response of hepatic (E) and peritoneal lesions (F) under therapy. (G) Graph shows values of γGT (left Y-axis) and CEA (right Y-axis) over time. Symbols mark administration of the indicated therapy. (H) Graph shows the daily administered dose of selected antiemetics. (I) Graph shows the volume of ascites drained every 48 h hours. (I, H) Vertical dotted lines indicate hospital admission and initiation of EC + FOLFOX. Schemes were created using BioRender.

A 26-year-old male patient initially presented with diffuse lower abdominal pain, diarrhea, night sweats, and weight loss. Gastroscopy and colonoscopy were performed and revealed a rectal tumor. Initial histopathological evaluation of the tumor showed a high-grade adenocarcinoma. Computed tomography (CT) showed the stenosing primary tumor with extensive compression of the superior rectal vein, diffuse hepatic metastases, intra-abdominal lymphadenopathy, PC and pulmonary embolism (Figure 1A and B). Within two weeks from initial endoscopy, the patient developed partial bowel obstruction and recurrent ascites requiring repeated paracentesis and finally developed secondary peritonitis requiring antibiotic treatment. The patient was then referred to our tertiary care center for further management.

Upon presentation, the patient was awake, oriented, and responsive. He reported abdominal pain and nausea. Laboratory evaluation of ascites fluid confirmed the diagnosis of peritonitis and PC. Repeated CT imaging showed rapid tumor progression within a short 2-week interval. Therefore, antibiotic treatment was continued, followed by rapid first-line induction chemotherapy consisting of folinic acid, 5-fluorouracil, and oxaliplatin (FOLFOX). Furthermore, molecular pathologic testing was initiated. The tumor was discovered to harbor a BRAF^V600E^ mutation while being RAS wild-type and microsatellite stable on immunohistochemistry and genetic testing.

Four days after initiation of chemotherapy, the patient developed progressive bowel obstruction requiring nasogastric tube placement and laxative and prokinetic treatment in combination with parenteral nutrition. Ten days after initiation of chemotherapy, repeated CT showed progressive PC and reduced intestinal passage, indicating a paralytical bowel obstruction. At this point, the patient was unable to tolerate any oral intake, including liquids and medication. He presented with nausea, vomiting, intermittent abdominal pain, fatigue, and ascites. Due to the irresponsive, rapidly progressive disease and clinical deterioration of the patient, the decision to intensify first-line chemotherapy by combining with cetuximab targeting epithelial growth factor receptor (EGFR) and encorafenib inhibiting BRAF (EC) was made. Due to complete bowel obstruction, rectal (300 mg daily) and enteral application via nasogastric tube (300 mg daily) of encorafenib was planned because standard dosing was suspected to lead to subtherapeutic plasma concentrations of the drug (Figure 1C) (Supplementary Methods).

## Molecular tumor board

Sole BRAF inhibition has been shown to result in compensatory overactivation of the EGFR-pathway in mCRC due to feedback activation, necessitating combinatorial blockade of EGFR and BRAF.^6^ Recently, the phase III BREAKWATER trial investigating the addition of FOLFOX to EC in patients with untreated BRAF^V600E^-mutant mCRC showed an enhanced response rate and survival in comparison to standard of care chemotherapy.^7^^,^^8^

However, while being convenient for most patients, oral drug formulations may pose a challenge in patients with abdominal cancer manifestations due to tumor-associated mechanical or paralytical bowel obstruction.^9^ This is especially relevant as patients with CRC carrying BRAF mutations are often affected from PC.^10^^,^^11^

## Patient update

Due to the untested route of administration and the applied dose of encorafenib, which was higher than approved for treatment, we were especially concerned with monitoring the patient for the occurrence of adverse events. (Table 1) Cardiovascular adverse events associated with encorafenib treatment did not occur in our patient. Routine laboratory investigations showed no relevant hepatic or renal toxicity. Furthermore, we did not observe ocular or skin toxicity related to encorafenib. Of note, an increase of γ-GT (Common Terminology Criteria for Adverse Events [CTCAE] °4) and alkaline phosphatase (AP) was observed (maximum CTCAE °1) which began prior to therapy initiation, peaked 4 days after the first dose, and then declined but did not normalize. Due to the increasing trend prior to initiation of therapy and the rapid decline thereafter, γ-GT and AP elevations were attributed to hepatic tumor growth in accordance with imaging studies. TRAEs included relevant hematologic toxicity. During therapy, the patient developed CTCAE °3 anemia requiring the transfusion of one packed red blood cell concentrate. Furthermore, the patient developed CTCAE °4 neutropenia, which resolved after application of G-CSF. Thrombocytopenia was mild (CTCAE °1) and did not require treatment. No dose reduction was necessary.

**Table 1.** Overview adverse events (AEs).

AE	CTCAE grade	Consequence	Comment
**Neutropenia**	4	G-CSF stimulation	Likely therapy related
**Thrombocytopenia**	1	—	Likely therapy related
**Anemia**	3	Transfusion packed red blood cells	Likely therapy related
**Nausea/vomiting**	4	Antiemesis, nasogastric tube placement, prokinetic medication, parenteral feeding	Present prior to therapy, improvement under therapy
**Abdominal pain**	3	Analgesia (WHO step III)	Present prior to therapy
**Ascites**	3	Paracenteses	Present prior to therapy, improvement under therapy
**Fatigue**	3	Multimodal treatment including psychosocial support, psycho-oncology and physiotherapy after exclusion of further treatable medical conditions.	Present prior to therapy
**Increase γ-GT**	4	Monitoring	Present prior to therapy, improvement under therapy
**Increase AP**	1	Monitoring	Present prior to therapy, improvement under therapy

To assess if combined nasogastric and rectal administration resulted in sufficient serum levels of encorafenib, therapeutic drug monitoring was performed. To this end, serum samples were drawn prior to daily dosing (20 h after the prior dose) and after 2.5 h. After 9 days of nasogastric and rectal administration, the trough (C_min_) and peak (C_max_) concentrations of encorafenib were 19 ng/mL and 109 ng/mL, respectively. (Figure 1D). During the clinical development of encorafenib, the mean C_min_ and C_max_ concentrations were 12 ng/mL and 2,240 ng/mL, respectively, in patients with advanced melanoma who were treated with a dose of 300 mg/day within the COLUMBUS study.^12^

Therapy with daily encorafenib and biweekly FOLFOX + cetuximab was continued for a total of 8 weeks (4 cycles). Despite the initially detrimental disease progression with FOLFOX alone (Figure 1A), a partial response on laboratory and imaging studies and a stabilization of the clinical performance status could be observed under this treatment regimen. After initiation of FOLFOX+EC, on CT-imaging after 4- and 8-weeks, response of the hepatic manifestations and PC was observed (Figure 1E and F). Although it was not possible to define a representative target lesion, exemplary hepatic metastasis in segment VII demonstrated radiologic tumor shrinkage (a reduction in diameter greater than 50%), consistent with a partial response for this lesion. Tumor markers CEA and γGT values declined most likely due to the response of hepatic metastases (Figure 1G). Furthermore, we observed a reduced need for the on-demand antiemetic drugs metoclopramide and dimenhydrinate after initiation of therapy, although the patient still required a nasogastric tube and parenteral nutrition. (Figure 1H). Additionally, the volume of ascites fluid drained also decreased under treatment, improving to °2 ascites (Figure 1I). Taken together, these findings indicate a tumor response associated with relevant improvement in tumor related symptoms.

However, five weeks after start of FOLFOX + EC, the patient developed a recurrence of peritonitis with Candida albicans sepsis, requiring aggressive antibiotic and antimycotic treatment. Due to general clinical deterioration, a change in the goal of therapy was discussed with the patient and best supportive care was provided by an outpatient palliative care service. Unfortunately, the patent died 58 days after initiation of therapy.

## Discussion

In this single-patient pharmacokinetic study, we describe experimental rectal and enterogastric application of only orally available encorafenib in a patient with severe bowel obstruction and inability of oral drug intake.

Despite aggressive prokinetic and complementary supportive treatment as well as initiation of first-line chemotherapy consisting of FOLFOX, the patient exhibited rapidly progressing disease and associated symptoms of subtotal bowel obstruction (ie, excessive vomiting, reduced stool frequency, weight loss, and abdominal pain) that were refractory to conservative management.

Following an extensive discussion on the poor prognosis of peritoneal metastases in young patients with CRC carrying a BRAF^V600E^ mutation, our patient felt that he would like to intensify treatment, if possible, in hope to indue remission.^3^^,^^13–20^ In light of the BREAKWATER trial results and the clinical course, the decision to escalate treatment to FOLFOX+EC was made.^7^ However, due to subtotal bowel obstruction caused by PC, reduced or even absent absorption of only orally available BRAF-inhibitor encorafenib had to be assumed. Of note, encorafenib is extensively metabolized in the liver (88% of the dose).^21^ Hypothesizing that tumor compression of the superior rectal vein in our patient would favor systemic over portohepatic uptake of the drug, we performed rectal administration in addition to administration via nasogastric tube to achieve the higher pharmacologically active plasma levels of encorafenib.^22^

A split dose of 300 mg transrectally and 300 mg via the nasogastric tube resulted in approximately 20-fold lower peak plasma (C_max_) concentrations while still achieving a sufficient trough concentration (C_min_), as compared to standard oral dosing of 300 mg in the COLUMBUS trial.^12^ Most likely, this reflects the impaired intestinal absorption due to progressive bowel obstruction with a generally slower uptake via the rectal route than oral absorption. Still, it appears that rectal administration might compensate for the limited gastric absorption. For kinase inhibitors like encorafenib, efficacy is primarily driven by sustained C_min_ rather than transient peak levels. Thus, despite markedly reduced C_max_, the maintained C_min_ suggests that overall systemic exposure remains adequate to achieve a therapeutic effect. A limitation of this study in this context is the lack of plasma exposure data immediately prior to initiation of rectal encorafenib administration, as the patient received a total of five single oral doses before addition of rectal applications.

Regarding clinical efficacy, a decrease in tumor marker serum concentration was observed soon after the addition of FOLFOX+EC in the presented case. Response on imaging could be observed after 4 weeks. Although we cannot rule out that this initial response is due to the first cycle of chemotherapy given without targeted therapy, the rapid deterioration within the first two weeks hereafter and the effective serum concentration of encorafenib argue against it. Importantly, we also noted reduced need for antiemetics and ascites fluid drainage, highlighting the reduced symptom burden after treatment initiation.

Due to the experimental way of drug intake in this study, two important considerations should be highlighted. Firstly, the patient was closely monitored for occurrence of adverse events in an inpatient setting. Secondly, therapeutic drug monitoring was performed to exclude accidental overdosing. Generally, serious TRAEs (°3/°4) observed in the patient encompassed hematotoxicity, nausea and vomiting which were shown to occur at a higher rate in FOLFOX+EC when compared to chemotherapy alone.^8^ However, while some symptoms (nausea, ascites) decreased, other symptoms that existed before treatment initiation including vomiting, pain and fatigue persisted during the course of therapy and, hence, were considered rather disease than treatment related.

During treatment, the patient developed a severe infection with bacterial peritonitis and Candida sepsis, which eventually led to clinical deterioration and, finally death of the patient. While the patient had been treated for peritonitis prior to initiation of FOLFOX+EC, we cannot exclude that this °5 AE was therapy-related. The observed OS of 58 days in our patient was substantially lower than the reported median OS in the original BREAKWATER trial.^8^ However, the severe condition and coexisting risk factors of the patient in this case would have precluded inclusion into the trial and can therefore not be considered representative.

In conclusion, we show that combined rectal and nasogastric administration of encorafenib is feasible in patients with BRAF^V600E^-mutant rectal adenocarcinoma and bowel obstruction, resulting in systemic trough drug exposure comparable to that achieved with oral administration in patients without gastrointestinal dysfunction. Furthermore, we document objective and clinical response in a patient with highly aggressive, BRAF^V600E^-mutant rectal cancer with bowel obstruction due to PC treated with FOLFOX+EC.

## Supplementary Material

oyag103_Supplementary_Data

## Data Availability

All data are included in this article. Additional details are available from the corresponding author upon reasonable request.
