# Supplementary material for: Gastric and rectal administration of encorafenib with targeted chemotherapy against BRAF V600E–mutant rectal cancer with bowel obstruction
Source: Oncologist. 2026 Mar 31;31(5):oyag103. doi: 10.1093/oncolo/oyag103 (PMC13092128; doi:10.1093/oncolo/oyag103)
Supplement: oyag103_Supplementary_Data [file oyag103_supplementary_data.docx]

Gastric and Rectal Administration of Encorafenib with Targeted Chemotherapy against BRAF V600E–Mutant Rectal Cancer with Bowel Obstruction: A Case Study of Safety, Pharmacokinetics and Efficacy

Maximilian Alexander Funk^1-4^, Volker Heinemann^2^, Veit Bücklein^1,2,4^, Stefan Karl Alig^5,6^, Kathrin Heinrich^1-4^, Lena Weiss^1-4^, Victoria Krenmayr^1-4^, Benoit Blanchet^7^, Wolfgang Gerhard Kunz^8^, Sebastian Theurich^1-4^, Michael von Bergwelt-Baildon^1-4^, Julian Walter Holch^1-4^

## Supplemental Methods

### Ethics statement

As FOLFOX+EC was not approved at the time of treatment, off-label use and potential associated risks were thoroughly discussed with the patient. Written informed consent was obtained prior to initiation of therapy. Furthermore, the patient provided written consent for data collection, scientific evaluation of the case, and publication of the results.

## Rectal and nasogastric application of encorafenib

Encorafenib at a dose of 300mg analogous to the BREAKWATER trial protocol was initially applied orally, which resulted in immediate expulsion via the nasogastric tube. Furthermore, due to greatly reduced intestinal passage, we assumed significantly reduced intestinal drug uptake even if instantaneous vomiting could be avoided pharmacologically. Therefore, it was decided to prepare a liquid suspension of encorafenib and to apply one dose of 300mg through the nasogastric tube and an additional dose of 300mg rectally. For each dose, four hard capsules of encorafenib 75mg were opened by hand wearing personal protective gear and the powder was pooled within a capped plastic container. 20 ml of physiologic saline were then injected into the container through a syringe and the powder was suspended until no more sedimentations were visible. The suspension was then injected into the nasogastric tube followed by pinching of the tube for as long as tolerable to avoid immediate expulsion (max. duration of clamping 4-5h). The second dose was applied transrectally as enema.

### Therapeutic drug monitoring

Blood samples were collected at steady state nine days after the start of rectal administration. The plasma concentrations of encorafenib were measured using a validated high-performance liquid chromatography method coupled with tandem mass spectrometry. Calibration was linear within the 1–1000 ng/mL range. Intra- and inter-precision for the three internal quality controls (15, 75, and 300 ng/mL) were below 10.7%, with intra- and inter-accuracy ranging from 93.6% to 107.1%. The accuracy of the method was verified through an external quality assessment scheme provided by the Group of Clinical Pharmacology in Oncology (Unicancer, Paris, France) and Asqualab (Paris, France).
